# Primary retroperitoneal hydatid cyst with intraperitoneal rupture: a case report

**DOI:** 10.1186/s13256-022-03415-6

**Published:** 2022-05-25

**Authors:** Kais Fourati, Ahmed Tlili, Abderrahmen Masmoudi, Taher Laabidi, Hazem Ben Ameur, Salah Boujelben

**Affiliations:** 1 Departement of surgery, Habib Bourguiba Hospital, Sfax, Tunisia; 2 Faculty of Medicine of Sfax, University of Sfax, Sfax, Tunisia; 3 Department of Surgery, Mohamed Ben Sassi Hospital, Gabes, Tunisia

**Keywords:** Hydatid disease, Psoas muscle, Retroperitoneal mass, Case report

## Abstract

**Background:**

Hydatid disease is endemic in Mediterranean countries and most commonly occurs in the liver followed by the lung. A primary localization in the retroperitoneum is extremely rare.

**Case presentation:**

We report the case of a 29-year-old Tunisian patient presenting with progressive left flank pain and skin urticaria. On abdominal ultrasonography and computed tomography scan, a ruptured retroperitoneal hydatid cyst was diagnosed, which was confirmed by positive hydatid serology. The treatment consisted of resection of protruding dome. The evolution was favorable. No local recurrence was detected during postoperative follow-up.

**Conclusions:**

Primary retroperitoneal hydatid cyst is extremely rare and has uncommon presentation, but we should learn the keys to its diagnosis. In endemic regions, high suspicion for this disease is justified regardless of localization.

## Introduction

Hydatid cyst is a zoonosis caused mainly by the larval stage of the cestode worm *Echinococcus granulosus* [1]. Hydatid disease (HD) is endemic in Mediterranean countries and most commonly occurs in the liver (55–70%) followed by the lung (18–35%). Tunisia is considered the most endemic Mediterranean country with mean annual surgical incidence of 12.6 per 100.000 inhabitants [2]. Therefore, it has serious impacts on public health. A primary localization in the retroperitoneum is extremely rare, with a reported prevalence of 0.5–5% in endemic areas [3–5]. Because it has an uncharacteristic presentation with often multiple organ invasion, there is a very high risk of misdiagnosis and a serious risk of surgery. Also, this form is very difficult to manage. In fact, there is a serious lack of discussion in literature as to how to manage primary retroperitoneal hydatid cysts.

We report a case of ruptured retroperitoneal hydatid cyst in a 29-year-old patient. Attention is drawn to the difficulty in diagnosis and management of such a rare presentation. This study is reported in line with the SCARE criteria [6].

## Case presentation

A 29-year-old Tunisian patient, with no pathological history, presented with progressively worsening left flank pain and skin urticaria. He had no history of trauma, nausea, or vomiting. His medical history was not significant, in particular lacking history of prior surgery. The patient is a construction laborer living in Sidi Bouzid, a governorate in the center of the country. Examination showed tenderness in the left flank. Hematological tests showed slight anemia with leukocytosis (13430/mm^3^) along with hypereosinophilia at 640/mm^3^. C-reactive protein (CRP) was elevated to 140 mg/L.

**Fig. 1:**
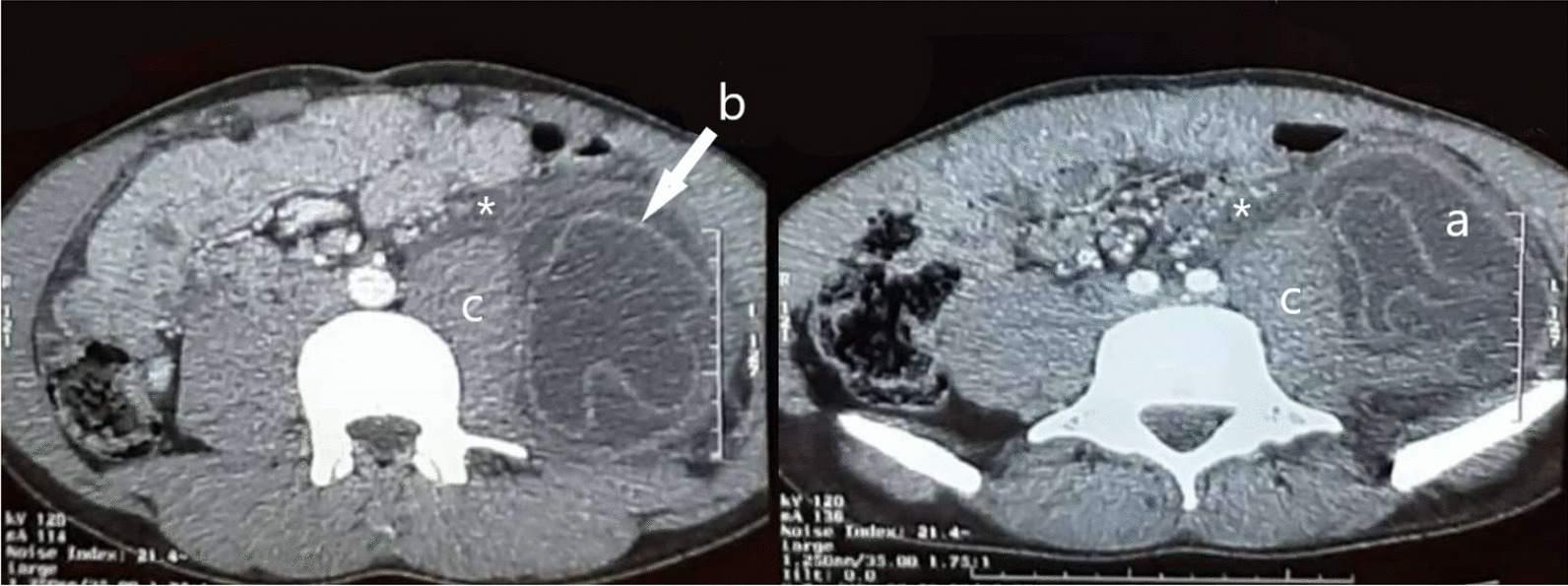
Computed tomography scan image showing hydatid cyst (**a**) with detached membrane **b** developed in the retroperitoneal space, adjacent to the psoas muscle **c** and associated to free peritoneal fluid*

Ultrasonography of the abdomen revealed a large cystic lesion in the left iliac fossa. Contrast-enhanced CT scan revealed a hydatid cyst of 12 × 7 × 21 cm^3^ with a detached membrane complicated with intraperitoneal rupture (Fig. 1). Because of the multiseptated aspect, it was classified as type II according to the Gharbi classification [7]. There was no evidence of similar cystic lesion in liver, lungs, or any other organ. Differential diagnosis included psoas abscess and malignant soft tissue tumor, but ELISA test for hydatidosis was positive. Therefore, based on the clinical, serological, and radiological evidence, a diagnosis of primary ruptured retroperitoneal hydatid cyst was made preoperatively.

**Fig. 2:**
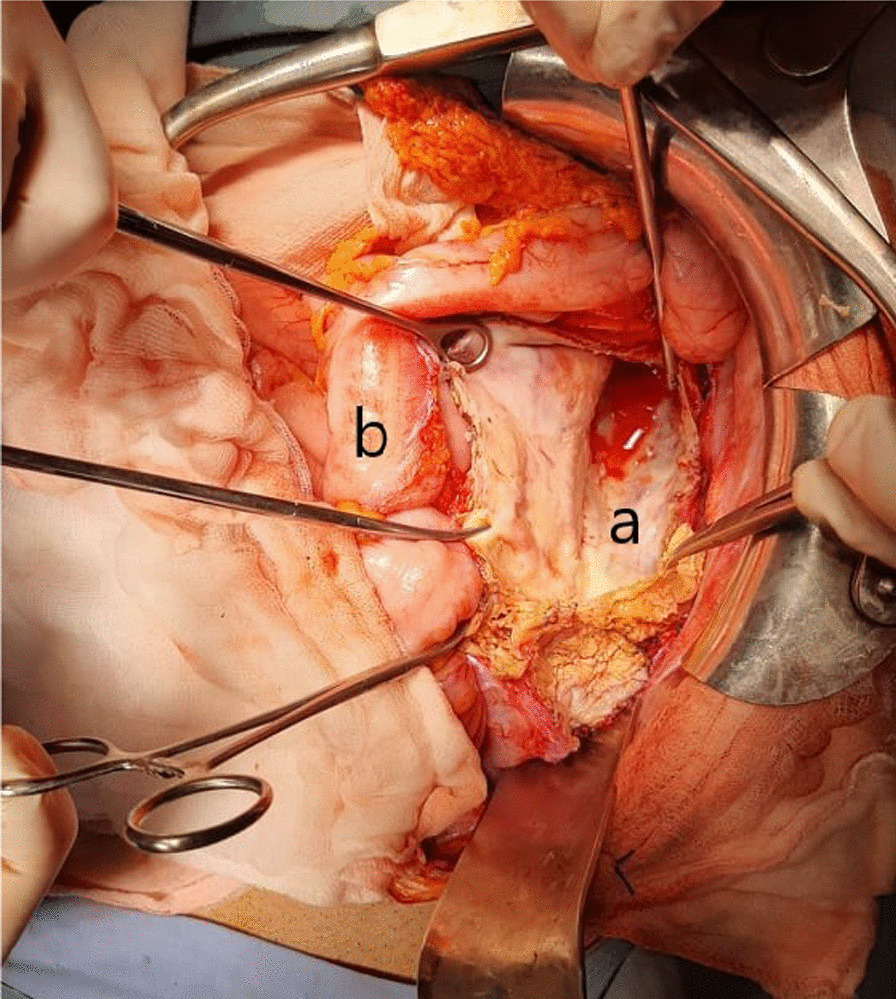
Intraoperative picture showing the disposition of the hydatid cyst **a** posterior to the left colon (**b**)

Exploratory laparotomy revealed a large cystic lesion with laminated membrane in the retroperitoneal space, extending from the lower pole of the left kidney down to pelvis, pushing the colon anteriorly and fissured in the peritoneum. We also identified four small daughter cysts along with peritoneal effusion of low abundance. The peritoneal cavity was washed out, then enucleation and partial cystectomy were carried out, observing the usual precautions (Fig. 2). Afterwards, the residual cavity was drained. The patient had an uneventful postoperative recovery and was discharged on postoperative day 3 on albendazole. Histopathology confirmed the diagnosis of hydatid cyst. No recurrence was observed after 1 year of follow‐up.

## Discussion and conclusions

Human is an accidental host in the life cycle of *Echinococcus granulosus*. When infested, about 95% of the larvae are trapped in the liver or the lung, while only 5% of them escape into the systemic circulation to involve other organs [8].

A primary retroperitoneal hydatid cyst is extremely rare [4, 5]. It is a distinct entity that must be considered when caring for a patient with a retroperitoneal mass in endemic regions [9]. In these cases, peritoneal involvement is seen in about 12% of cases [10], mainly because of secondary spillages due to rupture and other possible complications [3].

Clinical features include flank pain, abdominal mass, and nonspecific symptoms such as nausea and vomiting. Symptoms are usually due to compression as cysts increase in size [10]. When rupture or secondary infection occurs, acute symptoms arise. The differential diagnosis of a cystic retroperitoneal mass includes abscess, chronic hematoma, necrotic malignant soft tissue tumor, cystic lymphangioma, pancreatic cyst, and hydronephrosis [4, 11]. Diagnosis is established based on the combination of many parameters, including patient origin, clinical findings, imaging, and serology.

The radiological appearance of the hydatid disease of musculoskeletal system mimics tumors and other inflammatory conditions. Therefore, preoperative diagnosis is sometimes difficult clinically and radiologically [12]. Abdominal ultrasonography is a sensitive tool for diagnosing HC with characteristic findings such as floating membranes, hydatid sand, and daughter cysts. Presence of an undulating membrane and multiple daughter cysts within a mother cyst can suggest the diagnosis on CT and magnetic resonance imaging [13]. As in our case, when characteristic radiological findings are present, diagnosis can be confidently made with high specificity.

Surgery is the cornerstone for treatment of hydatid cysts [12, 14]. Total cystectomy without contamination of the field is the procedure of choice. As in our case, when total cystectomy is not possible because of dense adhesions to important anatomical structures, partial cystectomy should be done [15]. Furthermore, chemotherapy should always be considered in conjunction with surgery [16].

A retroperitoneal localization of the hydatid cyst is extremely rare. Rupture is a serious complication. The clinical symptoms can be confusing; therefore, preoperative diagnosis is sometimes difficult. In endemic regions, high suspicion for this disease is justified regardless of the localization affected, because delayed diagnosis increases the risk of impairment, recurrence, and sepsis.

## Data Availability

All data generated during the present study are included in the paper.

## Abbreviations

HD: Hydatid disease
CRP: C-reactive protein
CT: Computed tomography
